# COVID-19 Cross-Infection and Pressured Ulceration Among Healthcare Workers: Are We Really Protected by Respirators?

**DOI:** 10.3389/fmed.2020.571493

**Published:** 2020-09-10

**Authors:** Kenneth I. Zheng, Rafael S. Rios, Qi-Qiang Zeng, Ming-Hua Zheng

**Affiliations:** ^1^Department of Infectious Diseases, The First Affiliated Hospital of Wenzhou Medical University, Wenzhou, China; ^2^Clinical Research Center, The Second Affiliated Hospital of Wenzhou Medical University, Wenzhou, China; ^3^Institute of Hepatology, Wenzhou Medical University, Wenzhou, China; ^4^Key Laboratory of Diagnosis and Treatment for The Development of Chronic Liver Disease in Zhejiang Province, Wenzhou, China

**Keywords:** COVID-19, cross-infection, pressured ulceration, respirators, face mask

## Opinion

Anticipated vaccination development delay rendered non-pharmaceutical strategies crucial for the control of the coronavirus disease 2019 (COVID-19) pandemic. Thus, personal protective equipment (PPE) is the key for preventing potential amplification of nosocomial infection and viral transmission as demonstrated during the 2003 SARS outbreak (1). As of April 8 2020, 22,073 health care workers (HCWs) from 52 countries have reportedly been infected by the severe acute respiratory syndrome (SARS) coronavirus 2 (SARS-CoV-2) (2). This might imply that there is a certain limitation in the ability of PPE to protect HCWs from cross-infection. Also, it is surmisable that unequal access to PPE and limited instruction in its correct use, may drastically reduce its utility.

### Current Issues of PPE Among HCWs

**Figure 1:**
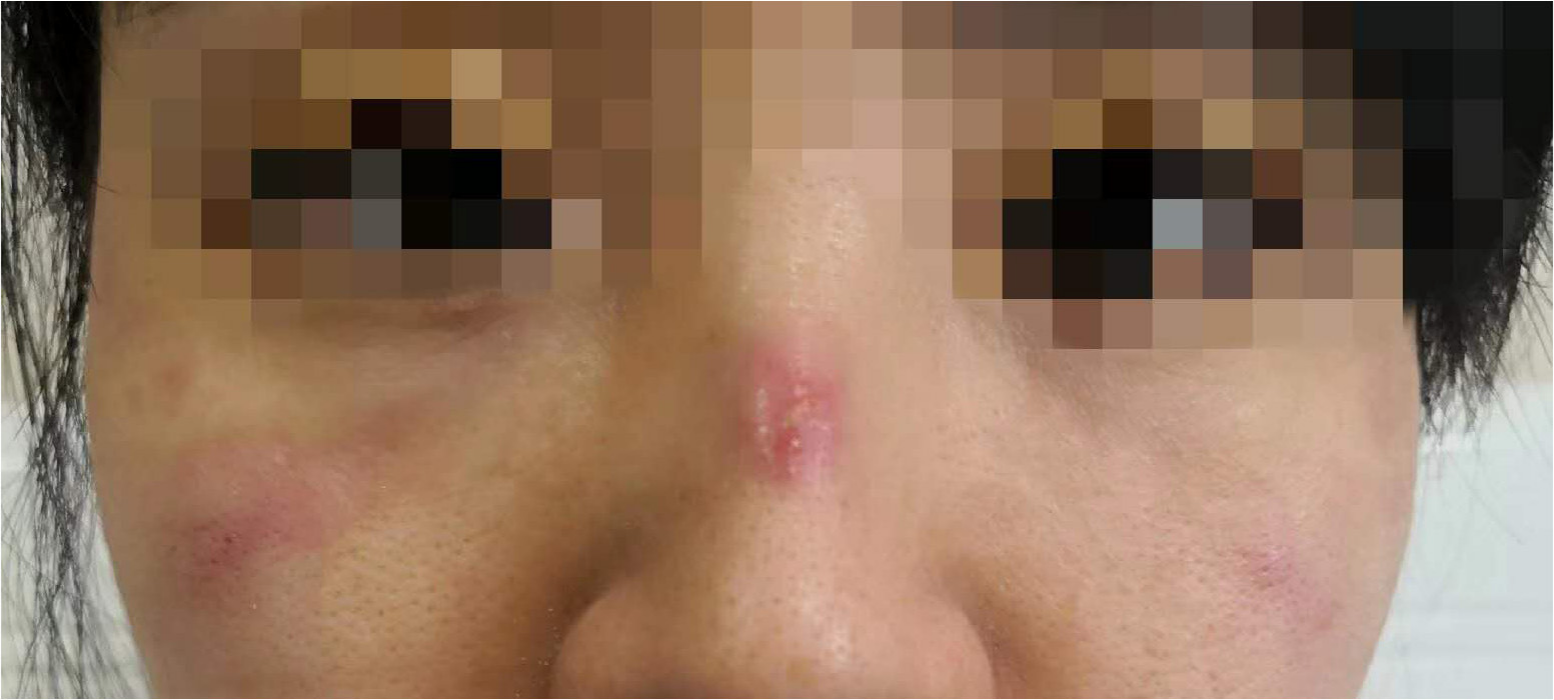
Photo taken of medical staff showing facial ulceration after prolonged use of a N95 respirator during the COVID-19 pandemic in China. Consent for picture publication has been granted.

For HCWs, the World Health Organization recommended the use of respirators including United States National Institute for Occupational Safety and Health (NIOSH)-certified N95, European Union standardized FFP2, or equivalently certified respirators, when performing or working in settings that require aerosol-generating procedures (3). The Occupational Safety and Health Administration respiratory protection regulations at 29 CFR 1910.134 mandated that HCWs be fit-tested and seal checked prior to the initial use of a respirator and whenever a different respirator face piece (size, style, model, or make) is used (4). However, not strictly following the aforementioned protocol and design flaws of current PPE are most likely what contributed to the high number of reported cases of SARS-CoV-2 infections among HCWs (5). In the current pandemic, although trained HCWs are highly adherent to strict protocols, they may not have access to respirators that fit him/her the best. Normally, a respirator should fit over the nose and under the chin with appropriate sealing through an arched metal nosepiece to prevent leakage. However, this is not always achieved due to variance in facial and nose bridge bone structure causing incomplete sealing. To combat this problem, HCWs may resort to manually shortening and fastening the straps that connect the respirator around the neck and over the head causing pressure ulcerations on facial skin along the edges of the respirator (Figure 1) (6). Even then, the safety of HCWs is not guaranteed. In a randomized clinical trial investigating the protection efficacy of N95 respirators (with daily usage of 5-h on average) in 949 HCWs, 37 (3.9%) had a clinical respiratory illness, while 13 (1.4%) had a laboratory-confirmed viral respiratory infection (7). In addition, 52.2% of users felt pressure on the nose, 41.9% felt uncomfortable, and 5% had skin rash. Preliminary analysis estimated the prevalence of skin damage from prolonged use (>6 h per day) of N95 respirator among 542 HCWs managing the COVID-19 pandemic in Hubei, China, to be 58.5% (8). Additionally, 526 HCWs reported skin damage due to respirator use, while most sites of skin lesions appeared on the nasal bridge (83.1%) and cheek (78.7%) (8). While it is obvious that the use of respirators can cause discomfort and harm to HCWs, there is a need to evaluate adherence of protocol and to assess the risk of infection from skin lesions due to PPE. Nevertheless, it is recognized that HCWs in the current COVID-19 pandemic, through prolonged wearing of respirators, might be exposed to a higher risk of cross-infection and skin damage.

### Potential Solutions

In order to reduce pressure ulcerations caused by prolonged respirator usage, relieving the pressure from the mask every 2 h was suggested by Gefen (6); however, this is not achievable, realistically, as hospitals are often short-staffed and HCWs must work around the clock to manage COVID-19 patients. The metal nose piece used to secure a respirator suggests a certain inadequacy in design. This implies the need for a better designed and improved respirator to strengthen its sealing capability and to reduce skin damage. One alternative solution to decrease facial pressure suggested was applying hydrocolloid padding along the sealing edges of respirators, creating a minute gap between the two (9). This technique may effectively lower the friction and chafing between PPE and the face, thereby drastically reducing skin lesions, although the integrity of the overall sealing is subject to further investigation. Recently, increasing evidence demonstrated the application of silicone foam dressing in reducing pressure ulcers (10, 11). The application of padding a double-sided silicone foam dressing on the inner surface of the respirator (along the nose arch) might provide a better seal between the face and edges of the respirator (Supplementary Figure 1). Both hydrocolloid padding and non-allergenic silicone foam dressing may reduce the facial pressure, while the latter is superior for reducing pressure ulcers incidents (any stage) and less prone to skin irritation (12). However, their applicability to PPE requires further investigation and testing.

In summary, the proper use of respirators among HCWs is pertinent for effectively preventing COVID-19 transmission. Unfitted and improperly fitted respirators are prone to leakage and may lead to an increased risk of SARS-CoV-2 cross-infection as well as pressure ulcerations in the skin, especially when used for a long time. It is necessary to improve the design of currently certified respirators in order to achieve better sealing capabilities and reduce pressure ulcerations.

## Author Contributions

M-HZ and Q-QZ: conception, design, and administrative support. KZ and RR: manuscript writing. All authors: final approval of manuscript.

## Conflict of Interest

The authors declare that the research was conducted in the absence of any commercial or financial relationships that could be construed as a potential conflict of interest.
