# Supplementary figures and images for: COVID-19 Cross-Infection and Pressured Ulceration Among Healthcare Workers: Are We Really Protected by Respirators?
Source: Front Med (Lausanne). 2020 Sep 10;7:571493. doi: 10.3389/fmed.2020.571493 (PMC7511704; doi:10.3389/fmed.2020.571493)

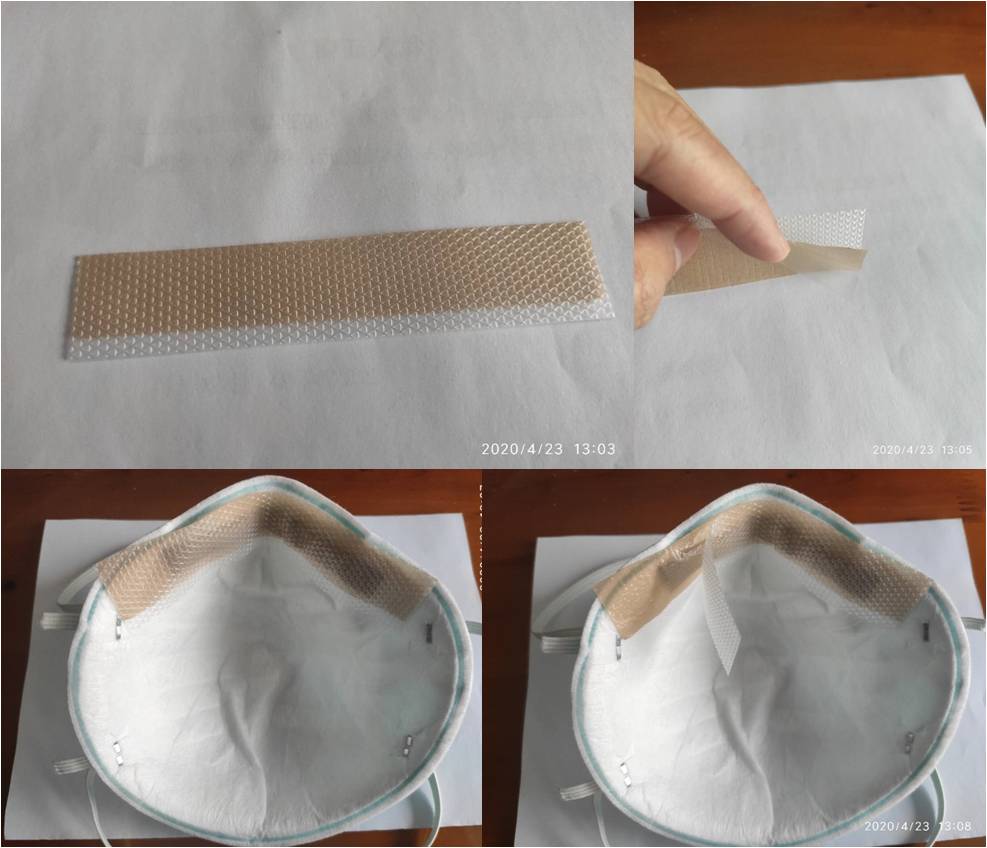

Supplement: Supplementary file 1 [file Image_1.JPEG]
